# Integrating NDVI, SPAD, and Canopy Temperature for Strategic Nitrogen and Seeding Rate Management to Enhance Yield, Quality, and Sustainability in Wheat Cultivation

**DOI:** 10.3390/plants13111574

**Published:** 2024-06-06

**Authors:** Oussama Hnizil, Aziz Baidani, Ilham Khlila, Nasserelhaq Nsarellah, Abdelali Laamari, Ali Amamou

**Affiliations:** 1 Research Unit of Plant Breeding and Genetic Resources Conservation, Regional Center of Agricultural Research of Settat, National Institute of Agricultural Research, P.O. Box 589, Settat 26000, Morocco; i.khlila@uhp.ac.ma (I.K.); nsarellah@yahoo.com (N.N.); 2 Laboratory of Agrifood and Health, Faculty of Sciences and Techniques, Hassan First University of Settat, P.O. Box 577, Settat 26000, Morocco; aziz.baidani@uhp.ac.ma; 3 Dryland Research Center, National Institute of Agricultural Research, P.O. Box 589, Settat 26000, Morocco; abdelali.laamari@inra.ma

**Keywords:** remote sensing, chlorophyll measurement, nitrogen efficiency, seeding strategy, yield, biomass, protein content, wheat growth

## Abstract

This study explores the interplay between nitrogen doses and seeding rates on wheat yield, biomass, and protein content. Utilizing tools such as the Normalized Difference Vegetation Index (NDVI), Soil Plant Analysis Development (SPAD) measurements, and canopy temperature (CT), we conducted experiments over five growing seasons. The treatments included three nitrogen levels (0, 60, 120 kg/ha) and three seeding rates (300, 400, 500 seeds/m^2^) in a split-plot design with 90 plots and two replications. Our results show that an intermediate nitrogen dose (60 kg/ha) combined with a moderate seed rate (400 seeds/m^2^) enhances wheat yield by 22.95%. Reduced nitrogen levels increased protein content, demonstrating wheat’s adaptive mechanisms under nitrogen constraints. NDVI analysis highlighted significant growth during the tillering phase with high nitrogen, emphasizing early-stage nutrient management. SPAD measurements showed that early nitrogen applications boost chlorophyll content, essential for vigorous early growth, while CT data indicate that optimal nitrogen and seed rates can effectively modulate plant stress responses. As crops mature, the predictive capacity of NDVI declines, indicating the need for adjusted nitrogen strategies. Collectively, these findings advocate for refined management of nitrogen and seeding rates, integrating NDVI, SPAD, and CT assessments to enhance yields and promote sustainable agricultural practices while minimizing environmental impacts.

## 1. Introduction

In wheat farming, providing a detailed understanding of crop management practices, particularly nitrogen use and seeding strategies, is critical to advancing sustainable agricultural practices. These factors are integral to enhancing crop yields, plant health, and nutritional quality, profoundly affecting both the productivity and ecological sustainability of farming operations. Developments in precision agriculture technologies such as the Normalized Difference Vegetation Index (NDVI), Soil Plant Analysis Development (SPAD) measurements, and canopy temperature assessments have dramatically improved our ability to refine these vital agricultural inputs [1,2,3].

Durum wheat (*Triticum durum Desf.*) is a staple crop globally, known for its adaptability and nutritional value. Effective cultivation of durum wheat requires careful management of nitrogen (N) and seeding rates to optimize yield and quality. Nitrogen management is particularly crucial because of its essential role in plant growth and the associated environmental risks, including water pollution and greenhouse gas emissions. Carefully managing nitrogen applications helps not only to improve plant health, but also to minimize ecological damage [4,5]. Additionally, selecting appropriate seeding rates is vital as it directly impacts how plants utilize the available space and resources, influencing their growth patterns, yield outcomes, and overall quality. Advanced strategies for optimizing nitrogen management can significantly enhance the yield and environmental performance of wheat crops while minimizing negative impacts, such as nitrate leaching and the emission of nitrous oxide [4,5]. Moreover, the integration of seeding rates with nitrogen management practices is essential for achieving optimal agronomic and environmental outcomes in wheat production [6,7].

Employing NDVI, we gain real-time insights into plant health, enabling precise adjustments to farming practices that cultivate optimal growth conditions. NDVI provides detailed feedback on plant light absorption and reflection, indicating plant health and the effectiveness of our agricultural strategies [8]. This technique has been widely used for various crops, including wheat, to estimate yield and monitor growth stages [9,10]. Studies have demonstrated NDVI’s effectiveness in optimizing nitrogen management and reducing environmental impacts [11]. Additionally, NDVI measurements have shown strong correlations with wheat yield variability, supporting its use in developing new fertilization strategies aligned with crop needs throughout the growing season [12]. SPAD measurements complement these insights by providing an estimate of chlorophyll content, which correlates with nitrogen levels in plants, shedding light on their photosynthetic activity [13]. These technologies, including multispectral remote sensing and machine learning, contribute significantly to precision agriculture by enabling more accurate monitoring of crop status and productivity. This allows for more targeted and efficient management of resources, ultimately leading to improved yield and sustainability of wheat production [12,14].

This research employed a split-plot experimental design over five growing seasons, involving five durum wheat cultivars and 90 plots to examine the effects of different seeding rates (300, 400, 500 seeds/m^2^) under varying nitrogen levels (0, 60, 120 kg/ha), with each plot replicated twice. This design mimicked real-world agricultural conditions, providing robust data on crop responses. Our study explores the relationship between nitrogen dosages and seeding rates to identify optimal strategies for managing wheat crops effectively. By integrating data from NDVI, SPAD, and canopy temperature measurements, we aim to refine farming practices that not only boost yields, but also do so sustainably. This approach leverages the capabilities of precision agriculture tools such as UAV multispectral imagery and machine learning algorithms to precisely manage nitrogen application, thus enhancing both productivity and environmental stewardship. Investigations into the effects of nitrogen management practices on SPAD values and NDVI readings have demonstrated the potential of these tools in optimizing wheat production under various environmental conditions [1,14,15,16].

The goal of this study was to investigate how different nitrogen levels and seed rates affect wheat yield, biomass, and protein content, utilizing precision agriculture tools. We aimed to identify and refine agricultural practices that enhance crop performance at various growth stages while supporting sustainable farming objectives. Through this research, we intend to offer actionable recommendations that can help improve wheat farming efficiency and environmental sustainability, contributing to better food security and agricultural practices.

## 2. Materials and Methods

### 2.1. Study Site and Environmental Conditions

The research covered five growing seasons (S): 2016–2017, 2017–2018, 2018–2019, 2019–2020, and 2020–2021, and took place at the Sidi El Aidi facility of the National Institute of Agricultural Research (INRA) in Morocco. Precipitation data over the five growing seasons are provided in Table 1. Further soil and site-specific parameters are summarized in Table 2. Soil samples were collected and analyzed annually before planting, with the results averaged to provide an overall view of soil conditions. The wheat was grown in a rotation with a fallow period each year to avoid additional nitrogen input from previous legume crops in the experimental station and to maintain soil health.

### 2.2. Experimental Design, Agronomic Practices, and Genetic Material

Each experimental plot covered an area of 2.7 square meters, measuring 2.5 m in length and 1.08 m in width. The study employed a split-plot design with five durum wheat cultivars and 90 plots to examine the effects of different seed rates (300, 400, 500 seeds/m^2^) under varying nitrogen levels (0, 60, 120 kg/ha), with each plot replicated twice. Standard agronomic practices, including soil preparation, weed control, and irrigation, were implemented to ensure optimal growth conditions. Sowing occurred in mid-November using a Wintersteiger plot seeder (Wintersteiger, Ried im Innkreis, Austria). Nitrogen was applied in two stages: half at the tillering stage and the remaining half at the stem extension stage. Ammonium nitrate fertilizer with a 33.5% nitrogen content was applied according to the treatment levels specified. Pest management included the application of Vitavax Ultra (Dhanuka, Haryana, India) for seed treatment and Roundup^®^ TURBO (St. Louis, MI, USA) for pre-emergence weed control. Manual weeding was performed as needed to maintain optimal crop health and to eliminate other factors that can affect the main factor of the study.

Five durum wheat cultivars were used in the experiments: Karim, Nassira, Faraj, Luiza, and Itri. These cultivars were selected for their adaptability and performance under Moroccan conditions (Table 3). The data used in this study are the mean of varieties for each combination of nitrogen and seed rate to remove the varietal effect and the mean of all the growing seasons to remove the environmental effect and the year-to-year variation.

### 2.3. Methodology for Data Acquisition and Trait Analysis

Yield measurements were quantified by harvesting the entire grain from each plot, measured in grams over an area of 2.7 square meters, later standardized to kilograms per hectare to ensure uniformity across the study. Biomass assessments occurred shortly before harvest, with the entire above-ground biomass from each plot recorded in kilograms to reflect productivity. For the evaluation of the critical quality trait of protein content, we utilized the capabilities of Chopin Technologies’ Infraneo, a near-infrared spectroscopy (NIRS) instrument (Chopin Technologies, Villeneuve-la-Garenne, France). This device underwent routine calibration at the National Institute of Agricultural Research facility in Rabat to maintain measurement precision. We strengthened the validity of the NIRS assessments through cross-validation processes, which involved comparing the Infraneo readings with those from a FOSS Infratec NIR analyzer (Infratec, Dresden, Germany), carefully calibrated and operated at INRA Settat. The protein content analysis was conducted using 800 g of grain collected from the harvested yield of each plot.

This dual-analytical approach not only enhanced the robustness of our protein content analysis, but also allowed us to cross-verify our NIRS data against the gold-standard Kjeldahl method. Through this validation process, we ensured that our protein content measurements were both accurate and reliable. This strategy of employing dual verification methods reinforces the integrity and consistency of our findings, providing a solid foundation for our research conclusions.

To augment our understanding of the plant physiological responses and enhance the precision of our agronomic evaluations, we incorporated several measurement techniques.

**Figure 1:**
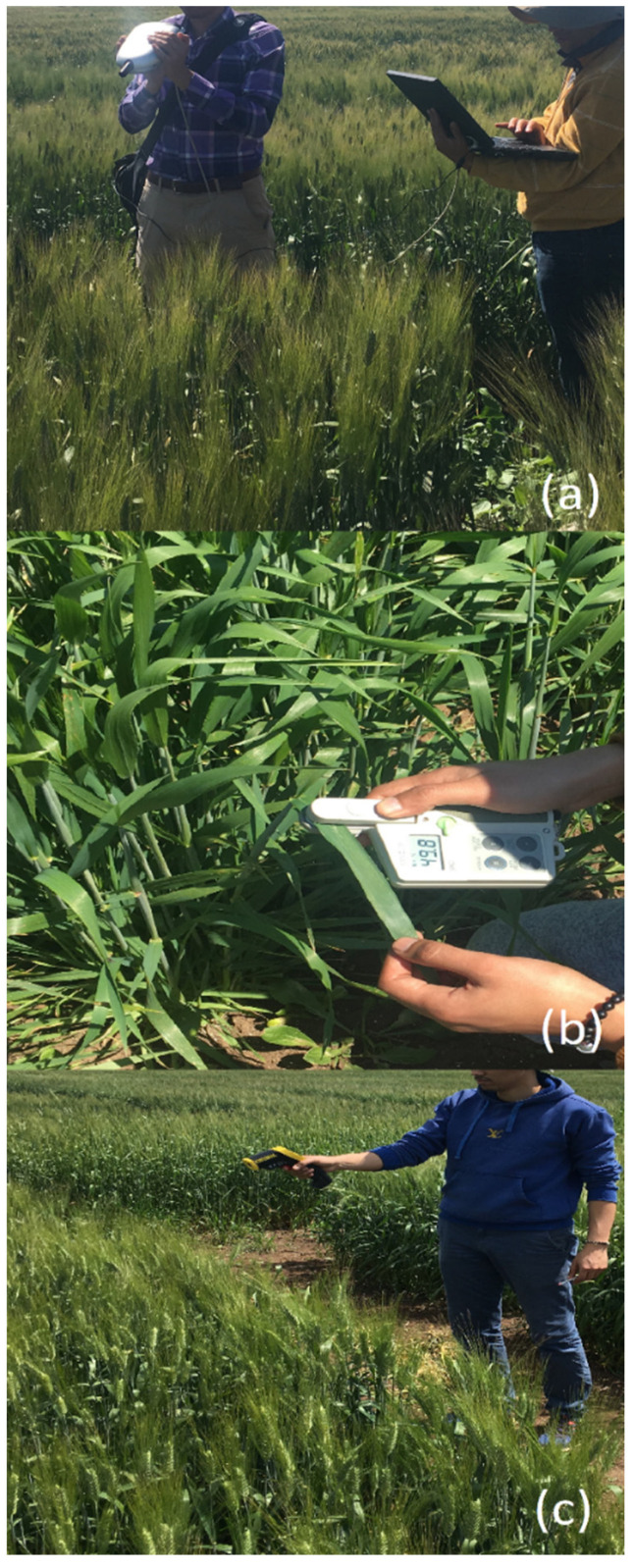
Implementation of precision measurement techniques in our field research. (**a**) Use of a multispectral radiometer for NDVI measurement. (**b**) SPAD-502 Plus meter used for assessing chlorophyll content. (**c**) Infrared thermometer for canopy temperature analysis.

Normalized Difference Vegetation Index (NDVI) measurements were performed using a FieldSpec HandHeld FSHH 325-3075P Spectris multispectral radiometer (Artisan Technology Group, Champaign, IL, USA). This tool measures reflectance at specific wavelengths to provide detailed insights into plant health and vigor. NDVI was calculated using the formula $NDVI=\frac{R900-R680}{R900+R680}$, where R900 is the reflectance at the near-infrared wavelength of 900 nm, and R680 is the reflectance at the visible light wavelength of 680 nm. Measurements were conducted during key phenological stages: tillering (Zadoks 20–26), stem extension (Zadoks 30–39), and heading (Zadoks 50–58) [17]. Each measurement was repeated ten times within each plot to account for variability, and the mean values were used for analysis. These measurements were taken under clear sky conditions between 10:00 a.m. and 2:00 p.m. to ensure consistency and minimize the impact of fluctuating light conditions Figure 1a.

Chlorophyll content, an indicator of plant health and nitrogen status, was measured using a SPAD-502 Plus meter (Konika Minolta, Tokyo, Japan). This non-destructive method allowed for rapid in-field assessment of chlorophyll levels providing a direct measure of the photosynthetic potential and nitrogen content of the plants. Measurements were conducted during the same key phenological stages as NDVI (tillering, stem extension, and heading). During each stage, measurements were taken from ten randomly selected plants per plot. From each plant, chlorophyll content was measured on the penultimate fully expanded leaf Figure 1b. Each leaf was measured three times, and the average value was recorded to ensure accuracy and consistency.

Canopy temperature was measured using an infrared thermometer to assess crop water stress and physiological responses. Measurements were taken under clear sky conditions between 10:00 a.m. and 2:00 p.m., providing data critical for understanding the thermal dynamics of the crop canopy and its correlation with water use efficiency and stress tolerance. For each plot, two measurements were taken from the part most exposed to the sun, ensuring the operator’s shadow and neighboring plots’ shadows were avoided. Measurements were consistently recorded from the same end of each plot with the sun behind the operator. The trigger was held for 3–5 s to allow the infrared thermometer to average the temperature readings (Figure 1c). Water stress in plants was assessed by comparing canopy temperatures between plots. Higher canopy temperatures indicate that the plants are experiencing more water stress due to decreased transpiration rates, leading to reduced cooling effects on the leaf surface. This method detects subtle temperature changes indicating stress levels based on plant physiology principles.

These methodologies, integrated into our study, enhance the robustness and precision of our agronomic assessments, enabling detailed analysis of how management practices influence crop performance.

### 2.4. Statistical Analysis Techniques

Data management began with the use of Microsoft Excel (version 2108, Build 14332.20324) for collecting all data annually for each trait studied and performing preliminary calculations, such as computing means, to prepare the dataset for further analysis.

For inferential statistics, we utilized Minitab 18 to conduct a two-way analysis of variance (ANOVA), which helped assess the significance of our findings and calculate standard deviations and F-values. This facilitated a deeper understanding of data variability and effect significance.

Following ANOVA, Tukey’s Honest Significant Difference (HSD) method was applied for post hoc tests to control the family-wise error rate and verify the distinctiveness of group means, ensuring rigorous comparison standards. The tests were performed at a significance level of 0.05. Minitab 18 was used to conduct the analysis, evaluating the effects of varying nitrogen doses and seeding rates. The results indicated mean values for each combination, accompanied by their respective letter annotations indicating statistical significance.

In addition, we employed R software (version 4.4.0) to perform Pearson’s correlation analyses, creating matrices that helped elucidate linear relationships between variables. 

To visually display our results, we used R to generate heatmaps, which visually represented the data’s variability through color gradients. Additionally, R was used to generate trend figures showing the NDVI and SPAD values across key growth stages under nitrogen doses. Origin Pro (version 2024b, 10.1.5.132) was used to produce boxplots that graphically depicted data distribution and central tendencies, offering insights into data spread. For the boxplot figures, significance levels were determined by one-way ANOVA and the subsequent Tukey’s test using Minitab 18.

## 3. Results

### 3.1. Analysis of Yield Responses to Nitrogen and Seed Rates

**Figure 2:**
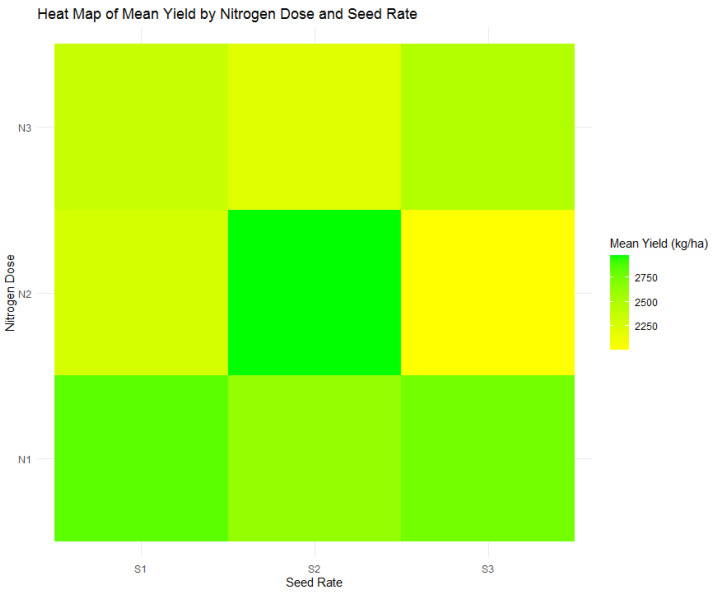
Heatmap of wheat mean yield variation by nitrogen dose (N1: 120 kg/ha, N2: 60 kg/ha, N3: 0 kg/ha) and seed rate (S1: 500 seeds/m^2^, S2: 400 seeds/m^2^, S3: 300 seeds/m^2^).

The agronomic experiment conducted provided an evaluation of wheat yield influenced by defined nitrogen doses (N1: 120 kg/ha, N2: 60 kg/ha, N3: 0 kg/ha) and seed rates (S1: 500 seeds/m^2^, S2: 400 seeds/m^2^, S3: 300 seeds/m^2^). Interactions between these variables on yield are graphically represented in Figure 2’s heatmap and quantitatively delineated in Table 4.

**Figure 3:**
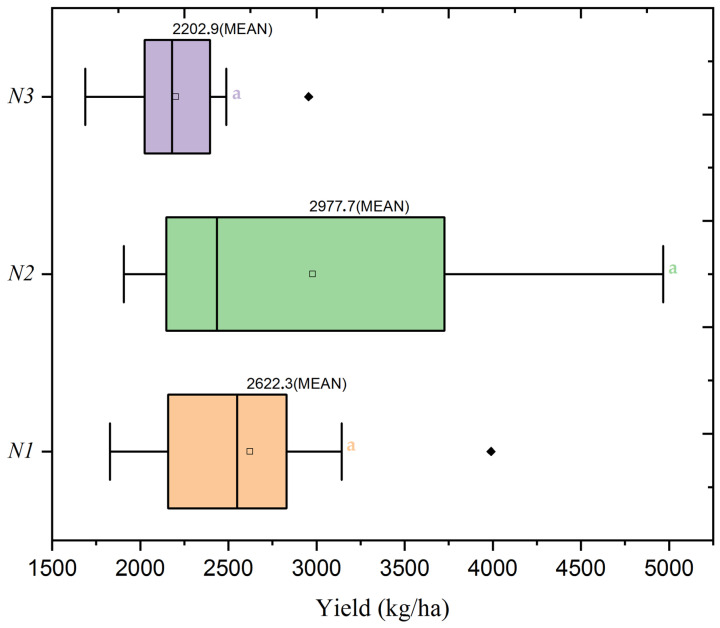
Boxplot of wheat yield distribution by nitrogen application levels (N1: 120 kg/ha, N2: 60 kg/ha, N3: 0 kg/ha) at the mean seed rate (S2: 400 seeds/m^2^). The black diamond symbol indicates outliers. Significance levels were determined by one-way ANOVA and subsequent Tukey test. Different letters indicate significant differences among the nitrogen application levels.

ANOVA results, presented in Table 4, highlight that nitrogen doses significantly influenced yield (F = 4.65, *p* = 0.012), while the seed rate alone did not have a significant impact (F = 0.92, *p* = 0.402). Moreover, the interaction between nitrogen dose and seed rate was significant (F = 4.38, *p* = 0.003), illustrating the crucial role of these combined factors in determining yield. As visually illustrated in the heatmap of Figure 2 and supported by the boxplot in Figure 3, this relationship was explored in more depth. The peak mean yield was achieved with the N2 dose (60 kg/ha) at a moderate nitrogen application rate, combined with the S2 seed rate of 400 seeds/m^2^, resulting in an optimal yield of 2978 kg/ha. This yield significantly surpassed the mean yield for the N2 dose by 22.95%, highlighting the effectiveness of this treatment combination. Conversely, the yield substantially decreased at the S3 seed rate with the same nitrogen dose (N2), yielding a mean of 2008 kg/ha—indicative of a 17.09% decline from the N2 average and a significant 32.58% fall from the highest observed yield.

The delineation of an optimal yield zone within the intermediate nitrogen dose range and a specific seed rate underscores the critical balance required in agronomic management. This balance is essential in directing the optimization of fertilization and seeding strategies for attaining maximum yield. These strategic insights have practical implications for promoting sustainable agricultural practices. 

### 3.2. Analysis of Biomass Responses to Nitrogen and Seed Rates

**Figure 4:**
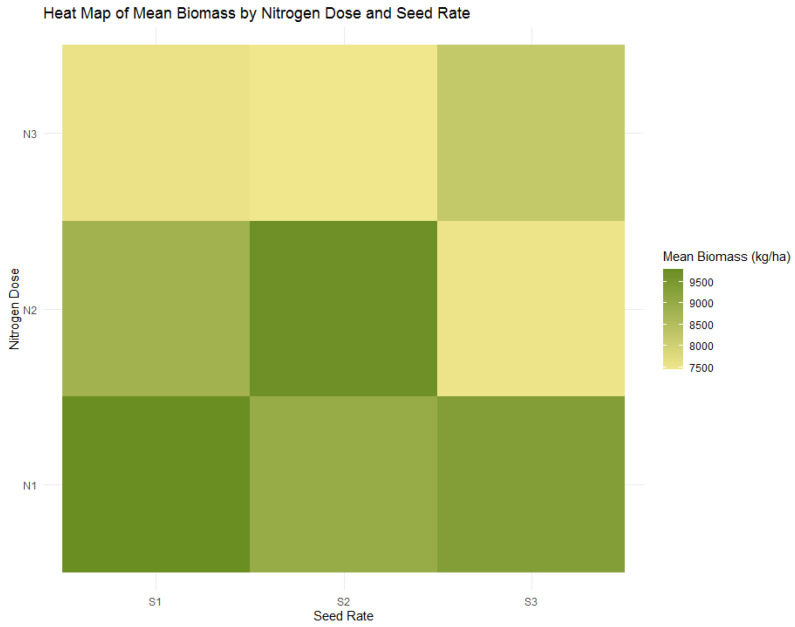
Heatmap of wheat biomass distribution by nitrogen dose (N1: 120 kg/ha, N2: 60 kg/ha, N3: 0 kg/ha) and seed rate (S1: 500 seeds/m^2^, S2: 400 seeds/m^2^, S3: 300 seeds/m^2^).

**Figure 5:**
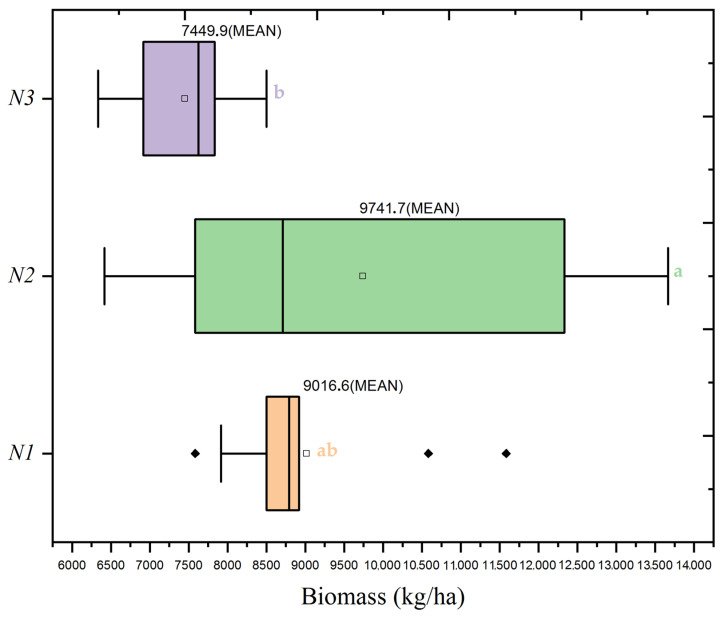
Boxplot of wheat biomass distribution by nitrogen application levels (N1: 120 kg/ha, N2: 60 kg/ha, N3: 0 kg/ha) at the mean seed rate (S2: 400 seeds/m^2^). The black diamond symbol indicates outliers. Significance levels were determined by one-way ANOVA and subsequent Tukey test. Different letters indicate significant differences among the nitrogen application levels.

The agronomic investigation conducted evaluated the dynamics of wheat yield and biomass, offering a global view of plant productivity. Both Figure 4 and Table 4 demonstrate that the agronomic treatments optimized yield and significantly increased biomass. The treatment conditions that produced the highest mean biomass further validated the effectiveness of the agronomic practices utilized. Specifically, the N2 dose paired with the S2 seed rate resulted in the highest biomass measurement at 9742 kg/ha, a 12.08% increase over the average biomass for the N2 treatments. This uniformity across different plant growth metrics implies the potential for a harmonized agronomic management approach. The results presented in Figure 4 and Figure 5 and Table 4 illustrate the impact of these agronomic strategies on productivity.

**Figure 6:**
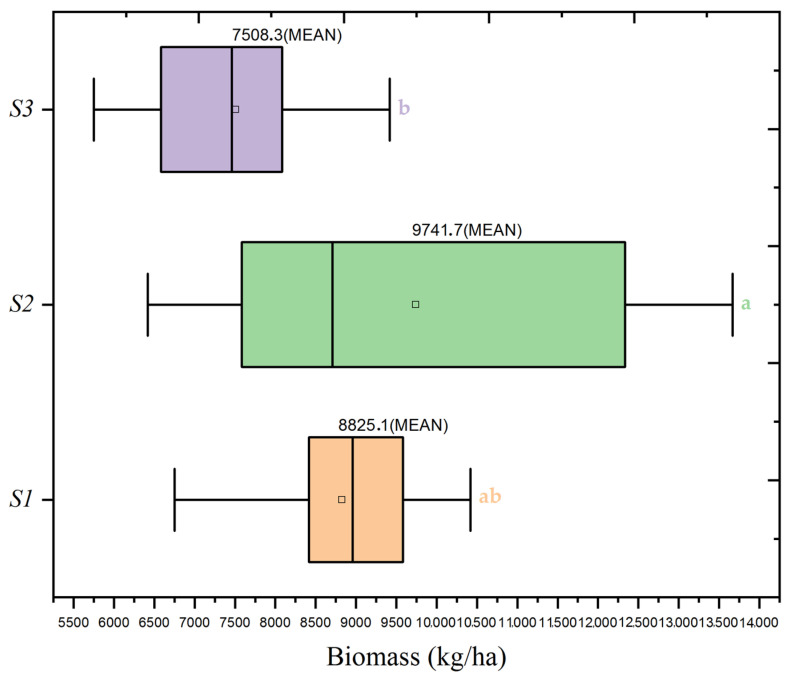
Boxplot of wheat biomass distribution by seed rate levels (S1: 500 seeds/m^2^, S2: 400 seeds/m^2^, S3: 300 seeds/m^2^) at a mean nitrogen application (N2: 60 kg/ha). Significance levels were determined by one-way ANOVA and subsequent Tukey test. Different letters indicate significant differences among the nitrogen application levels.

Figure 6 offers additional clarity on the influence of seed rates on biomass within the context of the N2 nitrogen dose. Here, the S2 seed rate under the N2 dose demonstrates a considerably higher mean biomass than S1 and S3, paralleling the yield trend. The mean biomass at the S2 seed rate was 9742 kg/ha, clearly indicating this seed density’s efficacy in maximizing biomass, akin to its impact on yield. However, the influence of seed rate on biomass is not significant when considered independently, as evidenced by an F-value of (F = 0.62, *p* = 0.539) It is within the interaction with the N2 nitrogen dose that the seed rate’s effect becomes significant, underscoring the importance of considering these factors in combination rather than isolation (F = 3.53, *p* = 0.01).

The findings portrayed in the boxplots are robustly supported by statistical evidence from Table 4, particularly the ANOVA results, which affirm the significant interplay between nitrogen doses and seed rates on biomass. While the impact of seed rate alone on biomass did not reach statistical significance, the interaction effects are substantial, reflecting the dynamics at play in crop production systems.

### 3.3. Analysis of Protein Content Responses to Nitrogen and Seed Rates

**Figure 7:**
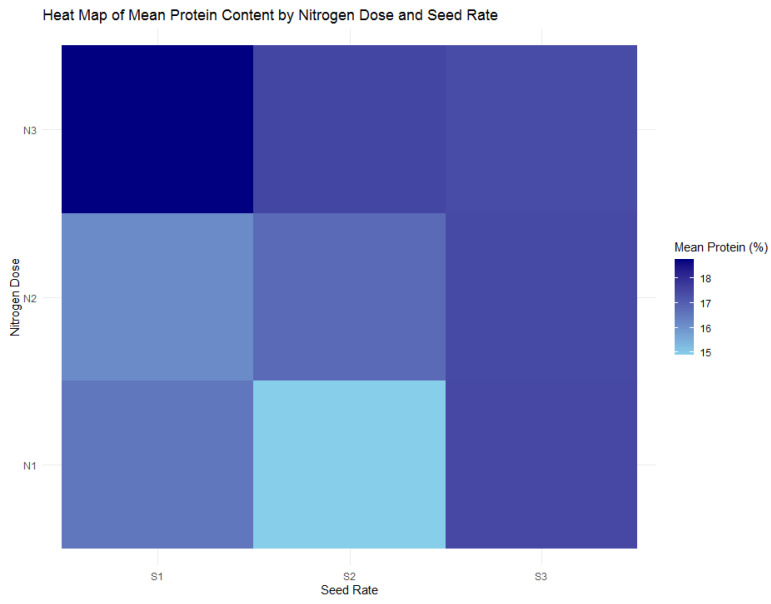
Heatmap of mean protein content in wheat by nitrogen dose (N1: 120 kg/ha, N2: 60 kg/ha, N3: 0 kg/ha) and seed rate (S1: 500 seeds/m^2^, S2: 400 seeds/m^2^, S3: 300 seeds/m^2^).

The analysis expanded from wheat yield and biomass to protein content to explore the variability of this essential quality parameter under varying nitrogen doses and seeding rates. Figure 7’s heatmap reveals contrasts in protein content across nitrogen doses, with the most pronounced protein content identified in the N3 group at an average of 18.25%. The ANOVA did not detect a significant interaction effect between nitrogen dose and seed rate on protein content, indicating that the observed differences are primarily due to the nitrogen dose alone.

**Figure 8:**
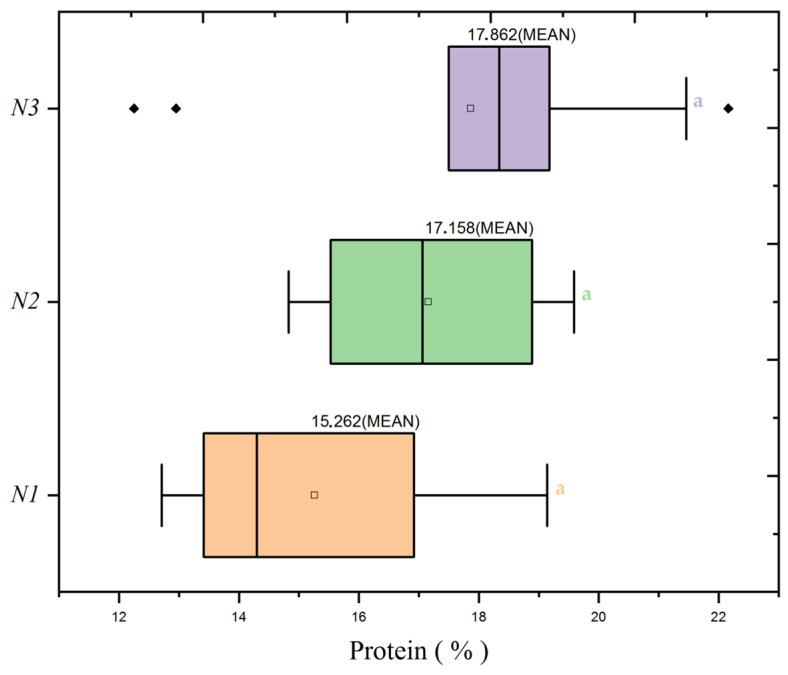
Variation of protein content in wheat as influenced by different nitrogen dose levels (N1: 120 kg/ha, N2: 60 kg/ha, N3: 0 kg/ha) at the mean seed rate (S2: 400 seeds/m^2^). The black diamond symbol indicates outliers. Significance levels were determined by one-way ANOVA and subsequent Tukey test. Different letters indicate significant differences among the nitrogen application levels.

**Figure 9:**
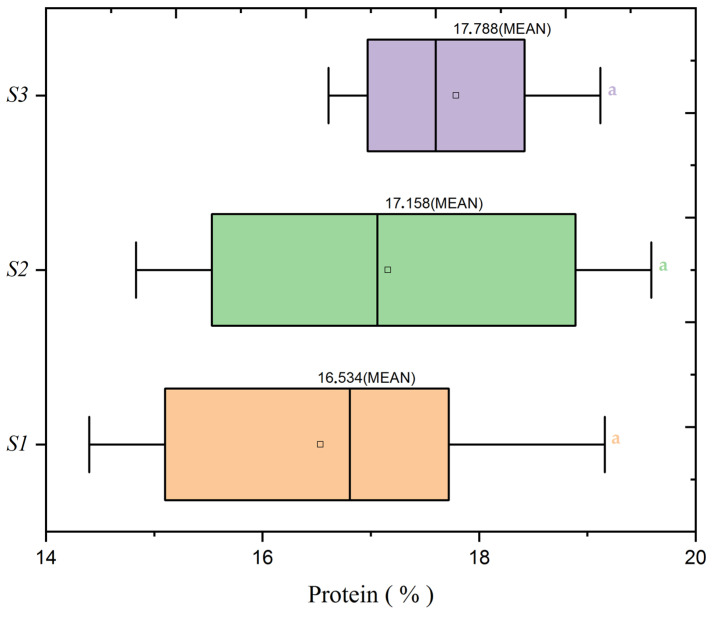
Protein content variability in wheat by seed rate levels (S1: 500 seeds/m^2^, S2: 400 seeds/m^2^, S3: 300 seeds/m^2^) at a mean nitrogen application (N2: 60 kg/ha). Significance levels were determined by one-way ANOVA and subsequent Tukey test. Different letters indicate significant differences among the nitrogen application levels.

Further investigating the protein distribution, Figure 8 demonstrates the effect of nitrogen doses, with the N3 treatment exhibiting a higher mean protein percentage than the N1 and N2 treatments. This trend suggests an adaptive response of wheat to limited nitrogen availability, which may represent a compensatory mechanism in protein concentration. Conversely, Figure 9 examines seed rate impacts and indicates that no particular seed rate significantly influences protein content.

The protein content analysis is statistically reinforced by Table 4 of the ANOVA results, which confirm a significant effect of nitrogen dose on protein content (F = 4.11, *p* = 0.02). While the seed rate alone did not show a significant impact (F = 1.79, *p* = 0.173), and neither did the interaction between nitrogen and seed rate (F = 1.88, *p* = 0.122), these outcomes hint at a latent complexity in how these factors influence protein content, indicating potential areas for future research.

The dataset indicates that optimizing protein content in wheat may align with reduced nitrogen input. This scenario contrasts with the conventional agronomic strategy of increasing nitrogen to boost yield. The data suggest that lower nitrogen doses result in higher protein content, challenging existing paradigms.

### 3.4. Evaluating the Impact of Nitrogen and Seed Rates on NDVI through Key Growth Stages of Wheat

The comprehensive investigation into wheat cultivation reported here expands the scope of traditional agronomic research by employing the Normalized Difference Vegetation Index (NDVI) to gauge plant health and photosynthetic capability at critical growth stages, thus offering essential insights for precision agriculture enhancements.

Statistical analyses from Table 5 confirm significant differences in nitrogen doses at both the tillering (F = 9.64, *p* < 0.001) and stem extension stages (F = 9.42, *p* < 0.001). However, the heading stage shows non-significant results (F = 3.09, *p* = 0.051), underlining a detailed relationship between plant development and nitrogen supply that demands additional investigation. 

Notably, seed rate alone did not significantly impact NDVI, suggesting that it may have a less dominant effect compared to nitrogen. However, the interaction effects observed at the tillering stage (F = 2.56, *p* = 0.045) point to a dynamic interplay between nitrogen and seeding rate, with the potential for diminishing influence as the crop matures.

**Figure 10:**
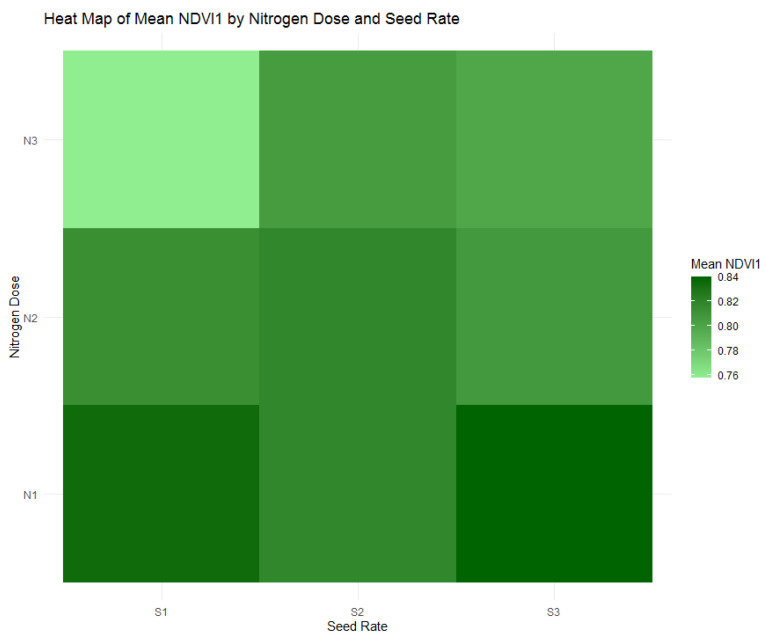
Heatmap of mean Normalized Difference Vegetation Index (NDVI1) by nitrogen dose (N1: 120 kg/ha, N2: 60 kg/ha, N3: 0 kg/ha) and seed rate (S1: 500 seeds/m^2^, S2: 400 seeds/m^2^, S3: 300 seeds/m^2^).

**Figure 11:**
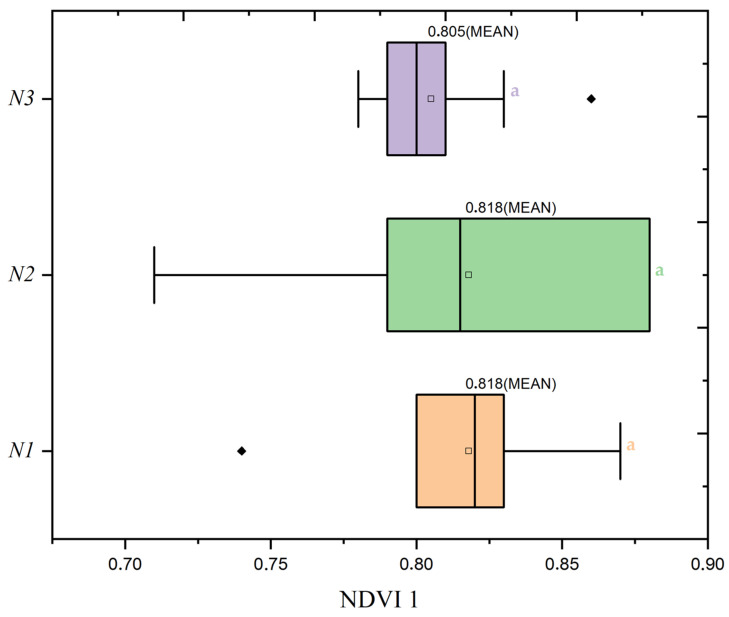
Distribution of Normalized Difference Vegetation Index (NDVI1) values by nitrogen dose levels (N1: 120 kg/ha, N2: 60 kg/ha, N3: 0 kg/ha) at the mean seed rate (S2: 400 seeds/m^2^). The black diamond symbol indicates outliers. Significance levels were determined by one-way ANOVA and subsequent Tukey test. Different letters indicate significant differences among the nitrogen application levels.

During the tillering phase, represented by NDVI1 in Figure 10, plants receiving the highest nitrogen dose (N1) of 120 kg/ha showed substantial growth across all seeding rates, particularly at the S3 seed rate (300 seeds/m^2^), underscoring the significant role of nitrogen in early plant development. This is further illustrated in Figure 11, where the distribution of NDVI1 values clarifies how nitrogen dose impacts plant health. Plants under the N1 treatment exhibited the highest NDVI1 values, indicating robust growth, whereas lower nitrogen doses showed decreased NDVI1 values, reflecting lesser vegetative vigor. 

**Figure 12:**
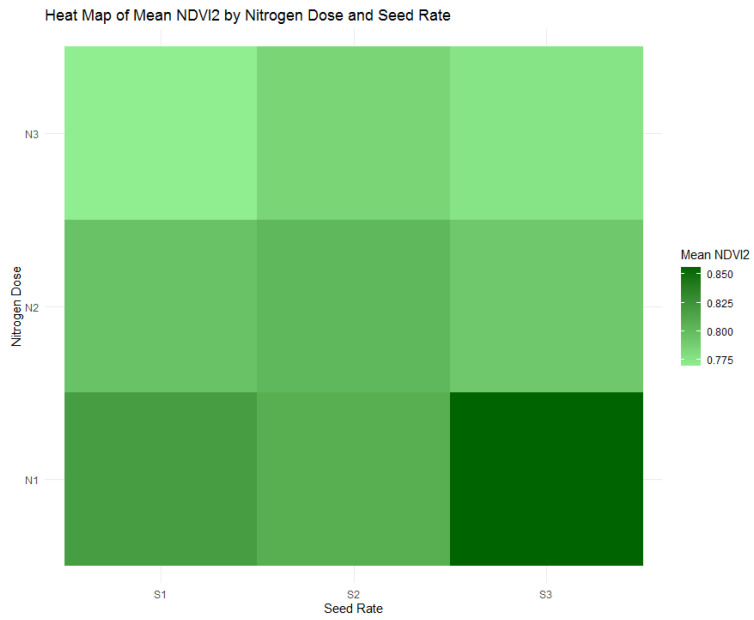
Heatmap of mean Normalized Difference Vegetation Index (NDVI2) by nitrogen dose (N1: 120 kg/ha, N2: 60 kg/ha, N3: 0 kg/ha) and seed rate (S1: 500 seeds/m^2^, S2: 400 seeds/m^2^, S3: 300 seeds/m^2^).

**Figure 13:**
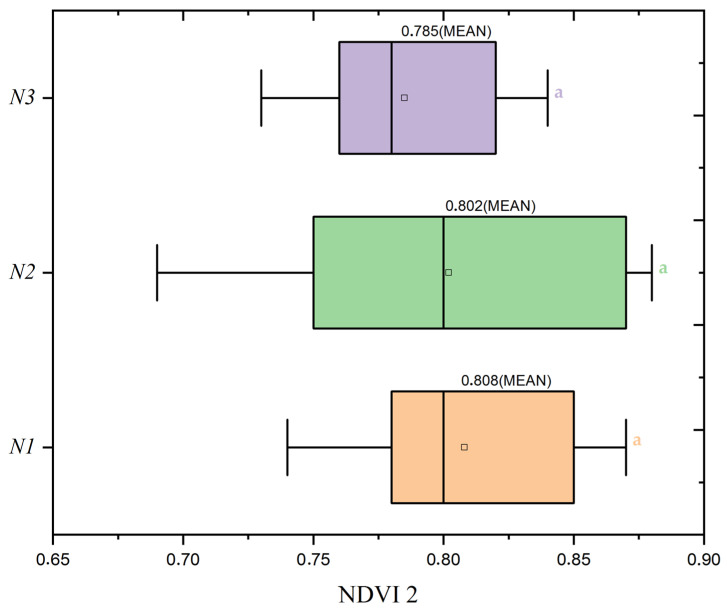
Distribution of Normalized Difference Vegetation Index (NDVI2) values by nitrogen dose levels (N1: 120 kg/ha, N2: 60 kg/ha, N3: 0 kg/ha) at the mean seed rate (S2: 400 seeds/m^2^). Significance levels were determined by one-way ANOVA and subsequent Tukey test. Different letters indicate significant differences among the nitrogen application levels.

As the study transitioned into the stem extension phase, reflected in NDVI2 with Figure 12 and Figure 13, the highest nitrogen dose group (N1) at 120 kg/ha maintained its position with the most consistent plant health, as evidenced by an average NDVI2 value of 0.827. This value suggests a continuation of robust vegetative growth from the tillering phase, indicating the sustaining effects of the highest nitrogen application during this crucial growth stage. Contrary to expectations of a reduction in vigor, this higher nitrogen availability underpins a stable vegetative health as the plants prepare for reproductive development. The data compel a reassessment of nitrogen management strategies to ensure plant health is optimized throughout all stages of growth, acknowledging the crucial role of nitrogen, especially in the early to mid-phases of the crop cycle.

**Figure 14:**
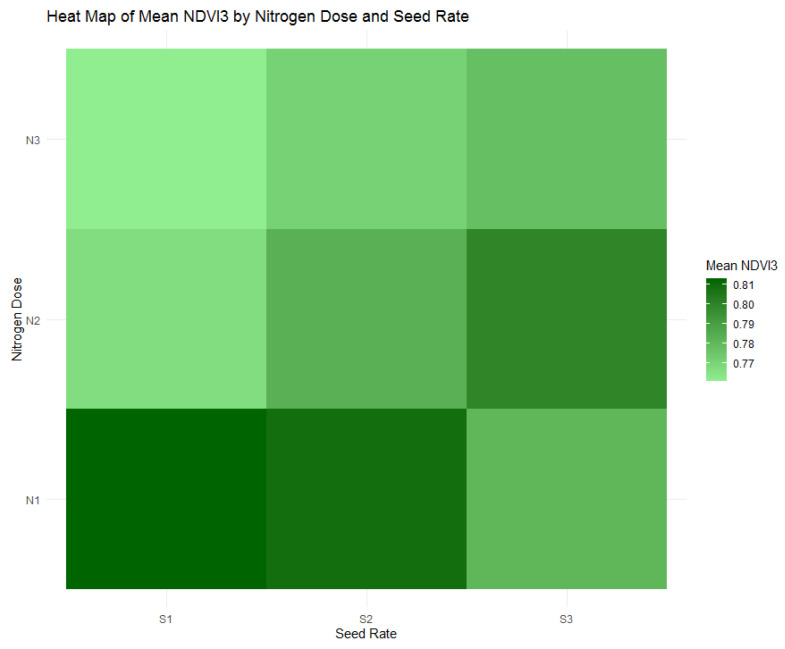
Heatmap of mean Normalized Difference Vegetation Index (NDVI3) by nitrogen dose (N1: 120 kg/ha, N2: 60 kg/ha, N3: 0 kg/ha) and seed rate (S1: 500 seeds/m^2^, S2: 400 seeds/m^2^, S3: 300 seeds/m^2^).

The data from Table 5, corroborated by Figure 14’s heatmap of mean NDVI3 values by nitrogen doses and seed rates, reveals a declining trend in NDVI3 across all nitrogen treatments as the wheat progresses to the heading stage. The highest nitrogen treatment (N1) group shows a mean NDVI3 value of 0.801, indicating a decrease from the tillering and stem extension phases. Similarly, the intermediate (N2) group exhibits a mean NDVI3 value of 0.784, and the non-fertilized (N3) group has the lowest mean value of 0.771, both confirming a general reduction in NDVI as the crops approach reproductive maturity. This trend reflects the anticipated shift in plant physiology from leafy, vegetative growth to grain filling, and suggests that the influence of nitrogen on vegetative vigor becomes less pronounced as wheat enters the reproductive phase. Table 5’s statistics provide a quantitative basis for this observation, underscoring the importance of adjusting nitrogen management strategies to align with the changing nutritional requirements of the wheat throughout its growth cycle.

**Figure 15:**
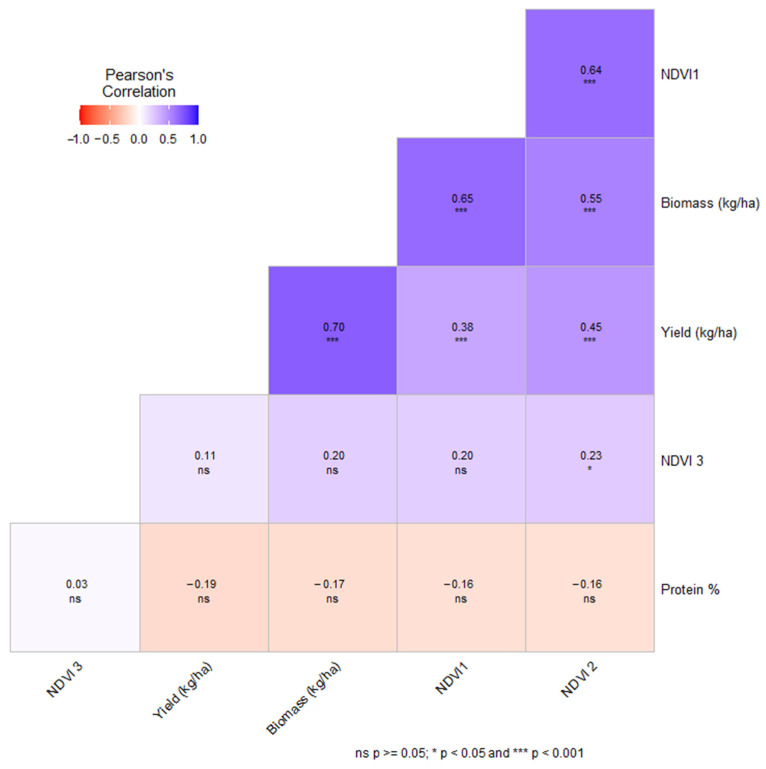
Pearson’s correlation matrix for wheat yield, biomass, Normalized Difference Vegetation Index (NDVI), and protein content.

In this study of wheat physiology, Figure 15 presents an insightful analysis of the interrelations among plant traits, particularly the Normalized Difference Vegetation Index (NDVI) during tillering (NDVI1), stem extension (NDVI2), and heading stages (NDVI3), and their influence on vital agronomic outputs such as yield, biomass, and protein content. 

The Pearson’s correlation coefficients showcased in Figure 15 reveal that NDVI1 has a strong positive correlation with biomass (r = 0.65, *p* < 0.001) and a moderate positive correlation with yield (r = 0.38, *p* < 0.001). These correlations confirm that a strong start in plant growth is indicative of higher biomass accumulation and a substantial influence on yield. Additionally, the positive relationship between NDVI1 and NDVI2 (r = 0.64, *p* < 0.001) suggests that early growth vigor is sustained into the subsequent stem extension phase, emphasizing the importance of initial nitrogen management.

In contrast, as plants progress to the heading stage, NDVI3 displays only a weak association with biomass (r = 0.20, *p* < 0.05) and an even weaker relationship with yield (r = 0.11, *p* < 0.05), indicating that NDVI’s predictive capacity diminishes as the wheat matures and transitions from vegetative to reproductive growth phases. 

Protein percentage demonstrates a lack of significant correlation with NDVI at all stages, suggesting that protein synthesis in wheat may be regulated by a set of factors distinct from those impacting vegetative growth. This finding is in line with the ANOVA results that show no significant effect of seeding rate or its interaction with nitrogen dose on protein content, revealing complex interactions that dictate this quality attribute.

The NDVI data derived from our study reinforce the critical need for an approach for nitrogen management throughout the wheat growth cycle. Our findings support a model of integrated management that optimizes both crop health and yield, promoting sustainable agricultural practices. This model emphasizes strategic nitrogen timing to enhance productivity efficiently while safeguarding environmental health.

The early-stage predictive capabilities of NDVI prove indispensable for agronomists, offering a method for the precise adjustment of agronomic inputs. These early indicators of plant health allow for the proactive management of resources, maximizing growth potential and ensuring resource use efficiency. This application of NDVI data not only improves yield outcomes, but also reduces the environmental footprint of agricultural practices by targeting input use where and when it is most effective.

Our research lays the groundwork for the next generation of farming practices that combine real-time monitoring with established agricultural methods to elevate crop yield. Looking ahead, leveraging NDVI within precision agriculture frameworks is set to transform crop management practices, ensuring they meet the dual objectives of enhanced productivity and environmental sustainability in an era of increasing global food demand.

### 3.5. Evaluating the Impact of Nitrogen and Seed Rates on SPAD Chlorophyll Measurements across Key Growth Stages of Wheat

Building upon our NDVI findings, we extend our agronomic analysis to the Soil Plant Analysis Development (SPAD) chlorophyll measurements, providing another dimension to our understanding of wheat physiology across developmental stages. The SPAD indices, like NDVI, serve as non-destructive proxies for assessing plant health, particularly chlorophyll content, which is closely related to nitrogen status and photosynthetic capacity.

Table 6’s statistical analysis reveals significant findings related to the influence of nitrogen dose and the interaction between nitrogen dose and seed rate on SPAD measurements, which are indicative of chlorophyll content in wheat leaves, a proxy for plant health and vigor.

For SPAD1, which reflects the chlorophyll content at the tillering stage, the nitrogen dose presents a statistically significant impact (F = 3.22, *p* = 0.045). This suggests that nitrogen application at this early growth stage is crucial for establishing the chlorophyll levels that support photosynthetic activity and initial plant development. The highest nitrogen treatment (N1) group exhibits the highest mean SPAD1 value, supporting the notion that adequate nitrogen supply is beneficial early in the plant’s life cycle and could guide targeted management practices to enhance early growth.

However, the significance of the nitrogen effect does not persist into the SPAD3 measurement stage (F = 1.65, *p* = 0.198), indicating that as the wheat plant progresses towards reproductive maturity, the direct influence of nitrogen on chlorophyll content becomes less pronounced. This observation suggests the need for a reassessment of nitrogen application strategies to ensure optimal resource utilization and support sustainable outcomes such as reduced nitrogen runoff and enhanced soil health.

Interestingly, the interaction between nitrogen dose and seed rate becomes statistically significant in the SPAD3 stage (F = 4.43, *p* = 0.003). This significant interaction effect at the heading stage implies that the combined influence of the amount of nitrogen available and the density of the seeding rate plays a more complex role in determining chlorophyll content during this later stage of development. It suggests that optimizing chlorophyll content, indicative of plant health during the heading stage, requires a balance between these factors, aligning agronomic practices with the plant’s changing nutrient demands.

**Figure 16:**
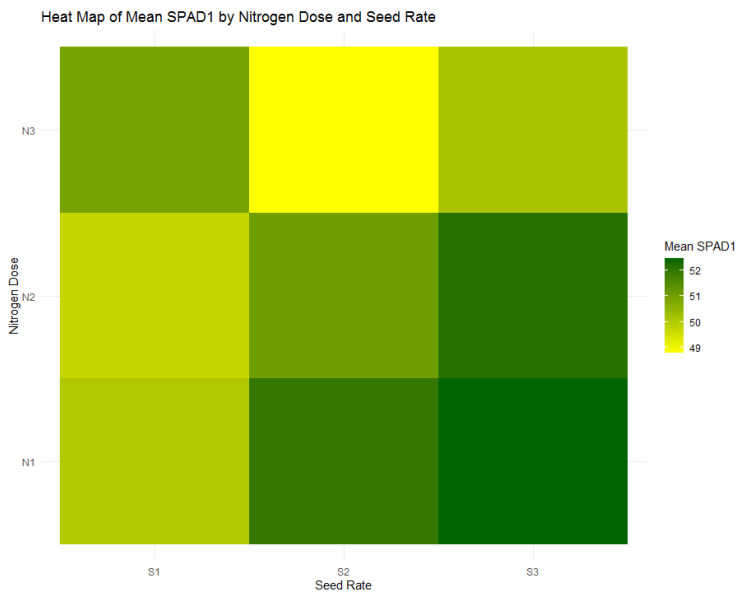
Heatmap of mean SPAD1 values by nitrogen dose (N1: 120 kg/ha, N2: 60 kg/ha, N3: 0 kg/ha) and seed rate (S1: 500 seeds/m^2^, S2: 400 seeds/m^2^, S3: 300 seeds/m^2^).

**Figure 17:**
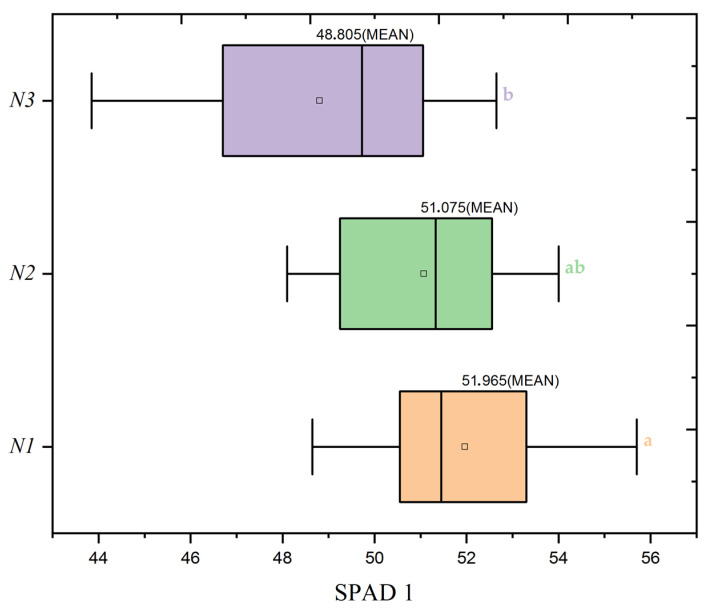
Distribution of SPAD1 values by nitrogen dose levels (N1: 120 kg/ha, N2: 60 kg/ha, N3: 0 kg/ha) at the mean seed rate (S2: 400 seeds/m^2^). Significance levels were determined by one-way ANOVA and subsequent Tukey test. Different letters indicate significant differences among the nitrogen application levels.

During the tillering stage, SPAD1 measurements indicate that plants receiving the highest nitrogen dose (N1) exhibit a pronounced chlorophyll content, reflected in an average SPAD1 value of 51.49. The heatmap in Figure 16 emphasizes that within the N1 group, it is the S3 seed rate (300 seeds/m^2^) that displays the highest mean SPAD1 reading of 52.47. This is consistent with the boxplot shown in Figure 17, which further confirms the superior performance of the N1 nitrogen dose within the medium seed rate category, highlighting the nuanced effects of nitrogen applications on chlorophyll levels during early growth stages. These findings align with NDVI1 observations, demonstrating the critical role of appropriate nitrogen provision in establishing vigorous plant growth, as posited by precision agriculture’s targeted nutrient management approach.

**Figure 18:**
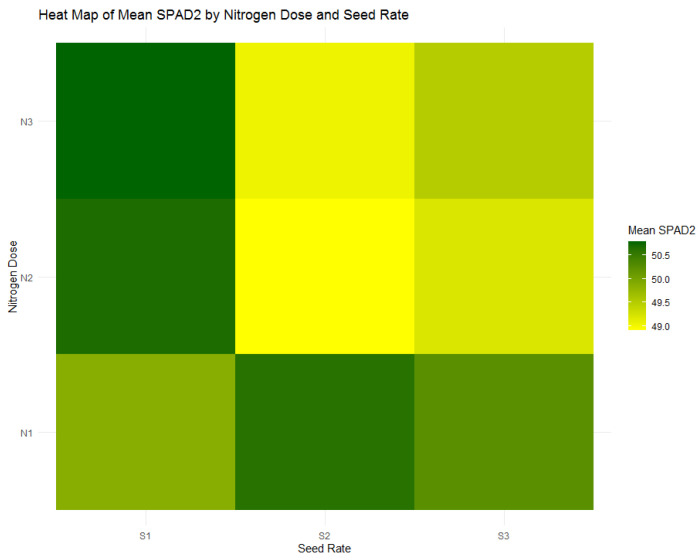
Heatmap of mean SPAD2 values by nitrogen dose (N1: 120 kg/ha, N2: 60 kg/ha, N3: 0 kg/ha) and seed rate (S1: 500 seeds/m^2^, S2: 400 seeds/m^2^, S3: 300 seeds/m^2^).

As illustrated by Figure 18, SPAD2 measurements at the stem extension stage indicate a consistent retention of chlorophyll content across nitrogen treatments. Specifically, the average SPAD2 value for the highest nitrogen dose (N1) slightly declines to 50.62, reflecting a continued healthy chlorophyll presence in the leaves. This persistence from the tillering to the stem extension phase underscores the critical role of early nitrogen application in maintaining chlorophyll density and, by implication, plant health throughout the vital early growth stages. 

**Figure 19:**
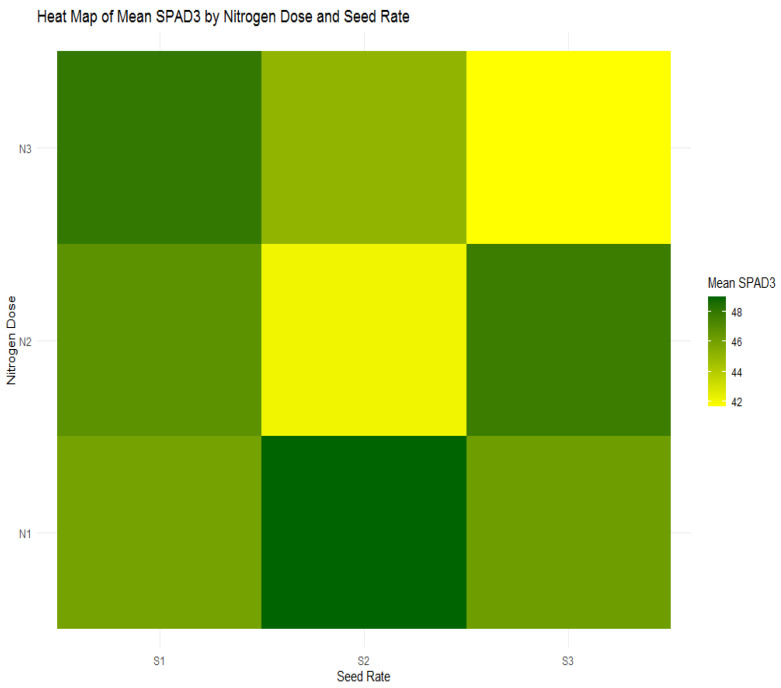
Heatmap of mean SPAD3 values by nitrogen dose (N1: 120 kg/ha, N2: 60 kg/ha, N3: 0 kg/ha) and seed rate (S1: 500 seeds/m^2^, S2: 400 seeds/m^2^, S3: 300 seeds/m^2^).

Advancing to the heading stage, Table 6 and Figure 19 indicates a decline in SPAD3 values across all nitrogen doses, mirroring the pattern observed with NDVI3. The highest nitrogen dose group (N1) shows a decrease to an average SPAD3 value of 47.06. This downward trend across the nitrogen treatments highlights a general reduction in chlorophyll content as the crop matures, transitioning resources from vegetative growth to grain filling.

**Figure 20:**
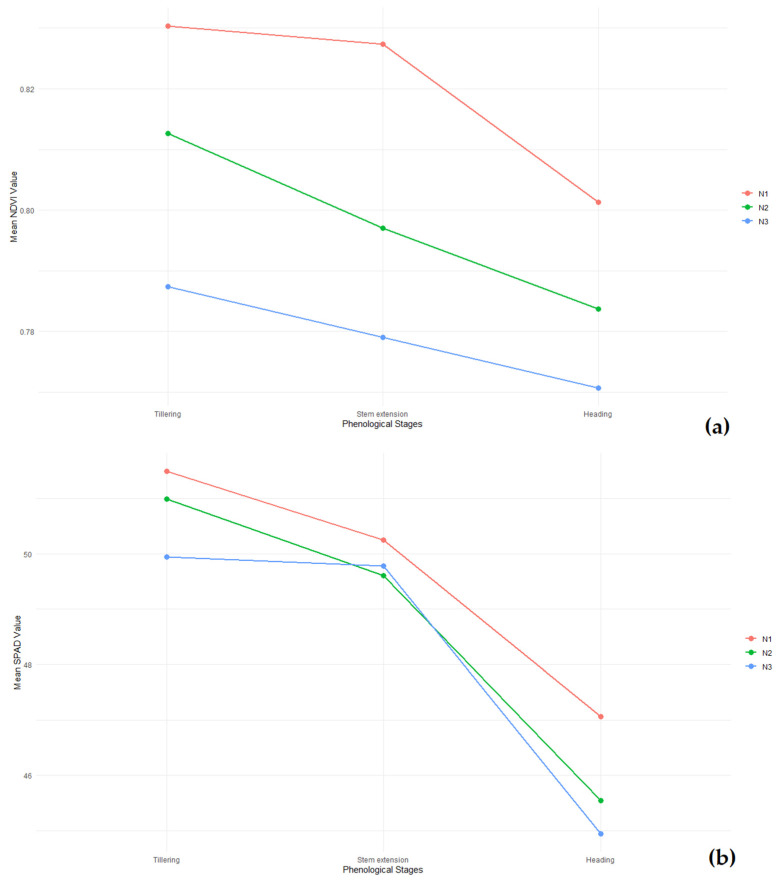
(**a**) Trends of NDVI values across key growth stages under different nitrogen doses (N1: 120 kg/ha, N2: 60 kg/ha, N3: 0 kg/ha). (**b**) Trends of SPAD values across key growth stages under different nitrogen doses (N1: 120 kg/ha, N2: 60 kg/ha, N3: 0 kg/ha).

To better visualize the trends of NDVI and SPAD values throughout the key growth stages, Figure 20 presents these indices over time. The NDVI values, as shown in Figure 20a, display a consistent decline from tillering to heading. The highest nitrogen dose (N1) maintains superior values throughout all stages, with the mean NDVI values for N1 starting at 0.830 during tillering and decreasing to 0.801 by heading. This trend emphasizes the importance of early-stage nutrient management, with statistically significant differences observed among nitrogen treatments (F = 9.64, *p* = 0.000 for NDVI 1; F = 9.42, *p* = 0.000 for NDVI 2; Table 5).

Similarly, the SPAD values, illustrated in Figure 20b, follow a declining trend but highlight significant differences among the nitrogen treatments, particularly at the tillering stage. The mean SPAD values for N1 start at 51.49 during tillering and decrease to 47.06 by heading, showing significant variation among treatments (F = 3.22, *p* = 0.045 for SPAD 1; Table 6).

In synthesizing these SPAD results with our earlier findings on NDVI, yield, biomass, and protein content, it becomes clear that nitrogen application must be managed strategically across the wheat’s lifecycle to promote not just yield, but also physiological health and efficiency. This approach advocates for precision in agronomic decision-making, emphasizing sustainable practices that optimize the various facets of crop development for high-quality agricultural production.

### 3.6. Impact of Nitrogen and Seed Rates on Canopy Temperature Dynamics in Wheat

Building on the insights from our NDVI and SPAD metrics analysis, we further explored the physiological responses of wheat to varying agronomic treatments by examining canopy temperature (CT) dynamics.

**Figure 21:**
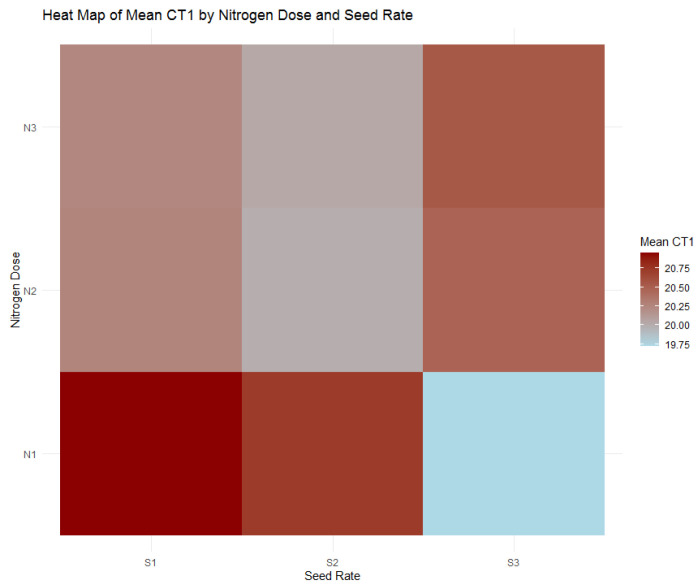
Heatmap of mean canopy temperature (CT1) by nitrogen dose (N1: 120 kg/ha, N2: 60 kg/ha, N3: 0 kg/ha) and seed rate (S1: 500 seeds/m^2^, S2: 400 seeds/m^2^, S3: 300 seeds/m^2^).

The relationship between canopy temperature (CT), seed rate, and nitrogen dose during the tillering stage reveals fascinating insights into wheat’s physiological response to varying agronomic practices. Table 7 highlights a significant interaction effect between nitrogen dose and seed rate on CT1 (F = 8.26, *p* < 0.001), elucidated by the corresponding heatmap in Figure 21. This interaction points to the balance between nitrogen availability and seed density that modulates canopy temperature, a proxy for water stress and photosynthetic efficiency.

The CT1 results demonstrate the effects of nitrogen and seeding rates on canopy temperature during wheat’s tillering stage. Specifically, the highest seed rate (S1) combined with the highest nitrogen dose (N1) registers a CT1 mean temperature of 20.95 °C, which aligns with expectations of a warmer canopy due to denser planting. On the other hand, a significant observation is that the same high nitrogen dose (N1) paired with the lowest seed rate (S3) results in the coolest mean CT1 temperature of 19.72 °C, as highlighted in Figure 21. This unexpected result may suggest a higher water use efficiency or a different physiological adaptation to thermal stress under these conditions. The distinct thermal footprint evident in the heatmap adds a layer of depth to our agronomic understanding and indicates that canopy temperature, much like NDVI and SPAD indices, can provide valuable insights into the optimal balance of agronomic inputs for healthy plant development.

As the study progressed to CT2 and CT3 measurements during the stem extension and heading stages, the statistical significance of the interaction between nitrogen and seed rate decreased (F = 2.07, *p* = 0.093 for CT2 and F = 0.33, *p* = 0.856 for CT3), as shown in Table 7. This trend underscores the necessity of adjusting nitrogen and seed rates in the early growth stages to effectively modulate canopy temperature, which could influence water use efficiency and overall plant health.

The findings from NDVI, SPAD, and CT measurements demonstrate that strategic management of nitrogen application and seed density throughout the wheat’s lifecycle is crucial for optimizing yield and ensuring plant health. This comprehensive approach promotes precision in agronomic decision-making and supports sustainable practices that enhance crop development and quality. By integrating precise agronomic data, we advocate a management model that enhances crop yield and sustainability, establishing a framework for agronomic excellence and environmental stewardship.

## 4. Discussion

In wheat agriculture, the significant interaction between nitrogen doses and seeding rates, as demonstrated in our study, corroborates the growing consensus on the need for nuanced nutrient management. Such a management strategy not only maximizes yield, but also enhances other aspects of crop performance. Specifically, we found that an intermediate nitrogen dose (N2) paired with a medium seed rate (S2) leads to optimal wheat yield, aligning with sustainable agronomy principles that advocate for precision rather than excess input use. This synergy reflects a complex balance, whereby nitrogen availability and seed density act together to optimize growth conditions, potentially minimizing environmental impacts. Our findings are supported by the work of Arduini et al. [18], who evaluated grain yield, and dry matter and nitrogen accumulation and remobilization, in durum wheat as affected by variety and seeding rate, illustrating how different seeding rates can impact wheat’s nitrogen use and yield under various conditions. Furthermore, the research by Malkarnekar Saharsh et al. [19] on the response of nitrogen and plant growth regulators on the growth and yield of wheat highlights the importance of appropriate nitrogen doses in conjunction with other agronomic practices to enhance yield and crop performance.

Additionally, Iqbal et al. [6] demonstrated that the interaction between seeding rates and nitrogen levels significantly affected grain yield, emphasizing the critical role of balanced nutrient management in achieving high yield. This finding supports our results and highlights the importance of optimizing both nitrogen doses and seeding rates for sustainable and productive wheat agriculture. Moreover, Ecco et al. [20] found that specific nitrogen doses positively influenced the number of tillers and spike length up to a certain threshold, although it did not significantly affect grain yield. These insights underscore the complexity of nitrogen’s impact on different wheat growth parameters and further reinforce the need for precise nutrient management strategies to optimize both nitrogen doses and seeding rates for sustainable wheat production.

Extending beyond yield, our study underscores the importance of considering crop biomass as a key output parameter. The highest biomass was recorded with the same N2 and S2 combination, suggesting that factors optimizing yield also benefit the overall plant growth, a relationship that has been previously observed. This dual benefit is critical, with biomass serving not only as a measure of crop productivity but also as an essential resource for livestock nutrition. Trentin et al. [21] demonstrate the significant impact of nitrogen fertilizer dosing on durum wheat biomass production, underscoring the importance of nitrogen availability in enhancing biomass and its potential benefits for livestock nutrition (Trentin et al. [21]). Additionally, the study by Souissi et al. [22] evaluates the effects of nitrogen fertilization on the agronomic and economic performances of durum wheat in rainfed semi-arid environments similar to those of our study. Their findings indicate that specific nitrogen dosages can significantly enhance N-use efficiency and grain yield, emphasizing the importance of nuanced nutrient management strategies in optimizing wheat biomass for enhanced livestock nutrition (Souissi et al. [22]).

Furthermore, the research by Latiri-Souki et al. [23] highlights that nitrogen fertilizer can increase dry matter, grain production, and radiation and water use efficiencies for durum wheat under semi-arid conditions, reinforcing the critical role of nitrogen in biomass production. Additionally, the study by Mon et al. [24] found that the interaction between nitrogen fertilization and irrigation significantly influences grain yield, canopy temperature, and nitrogen use efficiency, illustrating the complex dynamics between nitrogen management and environmental factors in enhancing biomass and overall crop performance.

The relationship between nitrogen levels and seeding rates sheds light on the nuances of agronomic practices. Although the combined effect of nitrogen dose and seed rate on yield was not statistically significant, it was observed that lower nitrogen rates, particularly when coupled with the highest seed rate, profoundly affected protein content, with the lower nitrogen dose (N3) surprisingly correlating with higher grain protein. This finding aligns with recent research by Melash et al. [25] and Banach et al. [26], suggesting that, under certain conditions, reduced nitrogen may lead to more efficient protein synthesis and accumulation. This phenomenon might be indicative of a nitrogen-sparing effect where the crop adapts to limited nitrogen by diverting it towards grain protein synthesis, presenting a case for re-examining nitrogen recommendations for protein optimization.

Contrary to our findings, Subedi et al. [27] reported that grain protein concentration generally increases with higher nitrogen application, even when grain yield did not benefit from split nitrogen application. This underscores the commonly accepted importance of nitrogen management in achieving high protein content. Similarly, Ghimire et al. [28] found that nitrogen application significantly increased grain yield and protein content, with spring and split applications showing better results than fall application in years with a risk of nitrogen loss. These studies suggest that, under typical conditions, higher nitrogen levels are expected to enhance protein content, whereas our study presents an interesting deviation where lower nitrogen levels coupled with higher seed rates resulted in higher protein content.

Moving beyond the tangible outputs of yield and biomass, our study leverages advanced agricultural tools to offer deeper insights into plant health and management strategies, such as NDVI, which has emerged as an invaluable tool in agronomy for assessing plant health and vigor across growth stages. Our study’s NDVI results offer compelling evidence of the role of early growth stages in determining the overall health and productivity of wheat crops. The pronounced growth observed during the tillering phase, under the highest nitrogen dose (N1), underscores the foundational importance of adequate nitrogen application for setting the stage for healthy plant development. This early vegetative vigor, as indicated by NDVI1, establishes a robust foundation that is strongly correlated with biomass accumulation (r = 0.65, *p* < 0.001) and has a significant impact on yield (r = 0.38, *p* < 0.001). Such findings corroborate the hypothesis that efficient nitrogen management at initial growth stages is paramount for optimizing subsequent crop outputs. Insights from relevant studies further reinforce the significance of precise nitrogen application as supported by NDVI data. The work of Vian et al. [29], demonstrates how NDVI, measured by an active optical canopy sensor, can be used to optimize nitrogen topdressing doses, enabling variable-rate nitrogen fertilization and affirming NDVI’s role in facilitating efficient nitrogen management. Additionally, Kizilgeci et al. [1] support the utility of NDVI in identifying nitrogen deficiency and enhancing precision nitrogen management in durum wheat cultivars under semi-arid conditions, showcasing its potential in ensuring food security amid climate change scenarios.

As the crop advances to the stem extension phase, the sustained high NDVI2 values reflect the continued vegetative health of the plants, highlighting the enduring effects of the initial nitrogen application. This phase marks a critical juncture where the maintained vigor from tillering supports the plant through its preparation for reproductive development. It is a testament to the necessity of reevaluating nitrogen application strategies to maintain plant health throughout the crop cycle, ensuring that the growth is not only sustained but also optimized for the reproductive phase. Li et al. [30] provide evidence supporting this notion. Their research indicates that adjusting nitrogen usage can significantly enhance grain yield and nitrogen utilization efficiency in wheat crops. Such findings suggest a crucial role of informed nitrogen management in sustaining plant health and optimizing production as the crop progresses through various developmental stages. Furthermore, Gezahegn et al. [31] demonstrated that split applications of nitrogen at specific growth stages significantly improved the yield and quality of durum wheat, reinforcing the importance of strategic nitrogen management throughout the crop cycle.

However, NDVI’s predictive capacity appears to wane as the plant transitions to the heading stage, evidenced by the declining NDVI3 values across all nitrogen treatments. This trend is indicative of the plant’s physiological shift from vegetative growth to reproductive development and grain filling, as demonstrated by Zhang et al. [32], who explored the impacts of optimizing fertilization on soil nitrogen cycling and wheat nitrogen utilization. Their findings underline the importance of adjusting nitrogen application to enhance wheat’s nitrogen uptake efficiency and reduce loss, shedding light on the relationship between nitrogen management and plant maturity stages [32]. The diminished correlation between NDVI3 with both yield and biomass as plants advance to the heading stage signifies a transition in plant physiology. This is further exemplified by Hussain et al. [33], who investigated how different nitrogen fertilizers impact grain yield and agronomic nitrogen use efficiency in wheat cultivars. Their work highlights how nitrogen source selection can significantly influence yield parameters and efficiency, emphasizing the necessity to tailor nitrogen management strategies to the specific developmental needs of the plant as it matures [33]. Additionally, Ibarra-Villarreal et al. [34] found that specific nitrogen rates can influence NDVI values and canopy temperature, further illustrating the nuanced impacts of nitrogen management on crop development.

Interestingly, the lack of a significant correlation between NDVI and protein content across all growth stages sheds light on the complex dynamics that govern grain quality traits. This disconnect suggests that factors influencing protein synthesis may extend beyond the vegetative growth parameters captured by NDVI, possibly encompassing a broader range of genetic and environmental influences. Studies by Dashkevich et al. [35], on the genetic potential of spring durum wheat, and by De Santis et al. [36], reviewing the influence of drought and abiotic stress on grain quality in Mediterranean environments, suggest such complexities. Dashkevich et al. revealed significant variations in protein content across durum wheat genotypes, underscoring the genetic underpinnings of quality traits, while De Santis et al. highlighted the role of environmental stressors in affecting protein content and composition, further emphasizing the multifactorial nature of grain quality. These insights indicate the profound implications for breeding and management practices, revealing the potential for targeted agronomic interventions to enhance grain quality without adversely affecting yield. Additionally, Mefleh et al. [37] found that genotypic variation in grain nitrogen content correlated with the content of different protein fractions, further emphasizing the genetic factors in protein synthesis. Afzal et al. [38] also highlighted the variability in protein profiles among wheat cultivars and the significant impact of environmental conditions on protein expression.

In synthesizing these insights, our study proposes a holistic approach to nitrogen and seed rate management that transcends traditional yield and biomass maximization objectives. By integrating NDVI-based assessments into agronomic decision-making, we can leverage early indicators of plant health to inform targeted interventions that enhance crop quantity. This strategy not only aligns with sustainable agricultural practices but also promotes environmental stewardship by optimizing input use and minimizing waste. The substantial economic benefits demonstrated by Finco et al. [39], through a decrease in labor and pesticide costs while enhancing nitrogen and seed distribution efficiencies, highlight the tangible outcomes of integrating precision agriculture in durum wheat production. Moreover, the potential for using NDVI as an effective tool for predicting yield and managing nitrogen applications in durum wheat is further supported by Wang et al. [40]. Their findings on crop models and canopy reflectance index, specifically NDVI at flowering time, as beneficial methods for managing nitrogen applications, echo our stance on the value of precision agriculture technologies. Together, these studies underscore the transformative potential of NDVI assessments and precision agriculture in fostering more nuanced crop management decisions, thereby advancing agronomic efficiency and sustainability in wheat production. Additionally, Toscano et al. [8] demonstrated the significant and positive linear relationships between NDVI and yield monitoring data, explaining most of the within-field variability in durum wheat, and highlighting the effectiveness of NDVI in precision agriculture. Vian et al. [29] also found that NDVI can be used to develop models for estimating shoot biomass and nitrogen content, which can help determine optimal nitrogen topdressing doses, thus improving nitrogen use efficiency.

Collectively, the findings underscore the transformative potential of integrating NDVI data into crop management strategies. This approach not only enhances our understanding of the dynamic interactions between nitrogen application, seed density, and plant growth, but also propels us toward more sustainable and efficient agricultural practices. Future research should continue to explore the multifaceted relationships between these agronomic factors and crop performance, expanding the scope of precision agriculture to encompass a broader range of crops and environmental conditions. The study by Santaga et al. [41] further supports this, showing that advanced precision nitrogen fertilization models limited yield losses and reduced intra-field variability under less favorable conditions. Mitra et al. [42] emphasized the effectiveness of NDVI sensor-based nitrogen management strategies in significantly improving wheat yield, nitrogen use efficiencies, and economic returns, validating the integration of advanced technologies in agronomic practices.

As we further explore the physiological aspects of wheat growth, the SPAD and CT results shed light on the complexity of plant health beyond conventional metrics. The SPAD results are indicative of chlorophyll content and, by proxy, plant health. The findings from Fiorentini et al. [43], who evaluated the relationship between SPAD readings, chlorophyll concentration, and Nitrogen Nutrition Index in durum wheat under various agricultural practices, demonstrated a strong association between SPAD readings, chlorophyll concentration, and efficient nitrogen management, supporting the essential role of nitrogen in establishing initial plant vigor, which correlates with overall plant productivity. Additionally, research by Sharma et al. [44] assessed the impact of different nitrogen and phosphorus fertilizer rates on chlorophyll content in winter wheat varieties, reinforcing the importance of nutrient management strategies for optimal plant growth reflected in SPAD values.

Moreover, the interaction between nitrogen dose and seed rate significantly impacts SPAD3 measurements, underscoring the nuanced nutrient utilization strategies as plants approach reproductive maturity. The findings from Skudra and Ruza [45] demonstrate that adjusted levels of nitrogen and seed rate markedly affect wheat growth and productivity, indicating the importance of these factors in enhancing chlorophyll content for sustained photosynthesis and grain development. This adaptation in agronomic practices, informed by precise nitrogen management and seeding strategies, is essential for maintaining plant vigor and optimizing wheat yield. Additionally, Jhanji and Sekhon [46] highlighted that chlorophyll meters could effectively quantify chlorophyll and nitrogen content in wheat leaves, showing significant correlations with the SPAD index, thereby enhancing the accuracy of nitrogen management practices. Furthermore, Wang et al. [47] found that multiple SPAD measurements on the same plants can improve the estimation of crop nitrogen status, further supporting the use of SPAD meters for effective nitrogen management in wheat.

As we examine the physiological intricacies of durum wheat development, the CT results from this study offer a fascinating glimpse into the thermal dynamics influencing plant growth. The observed coolest mean CT1 temperatures under conditions of high nitrogen application and low seed rate suggest an ideal thermal environment that may enhance growth due to reduced intra-species competition and optimized water usage. This observation resonates with the findings of Al-Karaki et al. [48]. Their work emphasizes the influence of thermal conditions on durum wheat, particularly how optimal temperatures can drive growth efficiency. Furthermore, as the wheat matures, the interaction between nitrogen and seeding rates diminishes, indicating a shift in the plant’s physiological needs. This phase of growth correlates with insights from Marti and Slafer et al. [49], who explored how durum wheat yields respond to a broad spectrum of environmental factors, highlighting the adaptive nature of plant responses to abiotic stress. Additionally, Cossani and Sadras [50] found that the interplay of nitrogen and water availability can influence how elevated temperature impacts wheat yield. Hou et al. [51] investigated the impact of experimental warming on nitrogen uptake in winter wheat, highlighting the significant effects of temperature and nitrogen management on crop performance.

In light of the relationships between nitrogen management, crop performance, and the incorporation of precision agriculture technologies, our study propels the discourse towards redefining conventional nitrogen management paradigms. The observed inverse relationship between reduced nitrogen availability and increased protein content necessitates a critical reassessment of nitrogen strategies. Such a phenomenon suggests that wheat possesses an inherent adaptive mechanism, potentially reallocating limited nitrogen towards grain protein synthesis under nitrogen-limited conditions. This insight, echoing findings from contemporary agronomic research, underscores the necessity for a recalibrated approach to fertilization that harmonizes yield optimization with grain quality enhancement. Abedi et al. [52] report that both the rate and timing of nitrogen fertilization play crucial roles in enhancing wheat protein content and its quality, alongside high wheat production, thus supporting a strategic rethink in nitrogen application strategies. Additionally, Li et al. [53] demonstrated that applying remedial nitrogen prior to low-temperature stress was more effective in enhancing wheat morphology and nitrogen uptake efficiency compared to post-low-temperature stress applications, highlighting the importance of strategic nitrogen management in varying environmental conditions.

The consideration of trade-offs in agricultural management—particularly between yield enhancement and protein concentration—highlights the complex balance required in crop management. This balance, reflecting a broader physiological response observed across crop species to varying nitrogen levels, advocates for a strategic approach to fertilization. Such an approach aims to balance nitrogen inputs to optimize both yield and grain quality, contributing to the evolving narrative on sustainable crop management practices. Recent studies, like that conducted by Karatay et al. [54], show the profitability and risk considerations of site-specific nitrogen management strategies in wheat production, which not only aim at enhancing grain quality and yield, but also consider price premiums associated with higher quality grains under different nitrogen management scenarios.

Furthermore, the integration of technologies such as NDVI and SPAD into our agronomic framework illuminates the path towards refined crop management strategies. By harnessing real-time insights into plant health and nitrogen status, these precision agriculture tools offer the potential to tailor agronomic interventions more closely to the plant’s lifecycle needs, thereby increasing both yield and quality outcomes. For instance, research by Kizilgeci et al. [1] highlights the effectiveness of SPAD and NDVI in assessing and optimizing nitrogen management strategies in durum wheat, demonstrating their critical role in improving grain yield and nutrient efficiency under semi-arid conditions. This supports the importance of these technologies in adapting agronomic practices to meet specific crop needs, enhancing sustainability and productivity in durum wheat cultivation.

Equally significant is our exploration of CT, which underscores the critical role of water use efficiency within the context of crop management. The strategic manipulation of agronomic factors to maintain optimal canopy temperatures shows the importance of adaptive management strategies in ensuring crop productivity, especially in water-scarce regions like the location of our study, Sidi El Aidi in Morocco. This facet of our findings reinforces the utility of precision agriculture in navigating the modern farming challenges, highlighting the importance of agronomic decisions in enhancing crop resilience and sustainability. In this context, the research by Devkota et al. [55] provides an analysis of genotype and agronomic management interactions that enhance wheat yield and water use efficiency in the Mediterranean rainfed environment of Morocco. Their study specifically examines how different genotypes perform under varied agronomic practices, shedding light on the optimal strategies to improve both yield and water use efficiency under the challenging conditions typical of Moroccan agriculture.

Our study underscores a crucial shift towards a more nuanced, stage-specific approach to crop management but also emphasizes the significance of integrating precision agriculture technologies to advance sustainable farming practices. By examining the adaptive responses of wheat to nitrogen availability and leveraging new technologies, we pave the way for future research aimed at fostering agricultural systems that address the pressing needs of food security while upholding environmental stewardship. As we continue to unravel the interplay between agronomic factors and crop performance, our collective efforts will be instrumental in shaping the future of sustainable agriculture. In particular, the study by Finco et al. [39] explores the economic outcomes of precision agriculture investments in durum wheat production, demonstrating how sustainable farming practices, combined with precision farming techniques, significantly enhance the sustainability and profitability of durum wheat cultivation, thereby supporting the broader goals of sustainable agricultural development.

## 5. Conclusions

This study examined the effects of various nitrogen doses and seeding rates on wheat yield, biomass, and protein content, employing precision agriculture technologies such as NDVI, SPAD, and canopy temperature measurements. Strategic nitrogen management, especially during the tillering phase as revealed by NDVI, is crucial for initiating robust early plant growth, which significantly impacts overall biomass and yield. The analysis demonstrated that an intermediate nitrogen dose combined with a moderate seed rate optimally boosts yield and biomass, showcasing the effectiveness of precision agriculture in refining crop management practices. NDVI data also highlighted a decline in vegetative vigor as plants matured, necessitating adjustments in nitrogen strategies to accommodate the plants’ evolving physiological needs. Furthermore, SPAD measurements confirmed that higher nitrogen levels substantially enhance chlorophyll content during early growth stages, supporting plant health. However, this influence wanes in later stages, indicating a physiological shift toward reproductive development. Canopy temperature data underscored that proper management of nitrogen and seed rates early in the growth cycle plays a vital role in modulating plant stress responses and enhancing water use efficiency. Together, these findings advocate for finely tuned nitrogen and seeding strategies to not only enhance crop yield, but also to promote sustainable agricultural practices. This integrated approach underscores the potential of precision agriculture to significantly improve both the efficiency and sustainability of wheat production, aligning advanced agronomic tools with sustainable farming objectives.

## Figures and Tables

**Table 1:** Research station’s soil characteristics, annual rainfall, geographic details, and elevation over five growing seasons.

Research Station	Soil Type	Annual Rainfall (mm) per Growing Season						Geographic Coordinates		Altitude (Meters)
		2016–2017	2017–2018	2018–2019	2019–2020	2020–2021	Mean Precipitations	Latitude	Longitude	
Sidi El Aidi (SEA)	Vertisol	290	505	210	242	467	343	33.12218°N	7.63315°W	235

**Table 2:** Soil and Site-Specific Parameters.

Parameter	Value	Classification
pH	8.25	Alkaline soil
Conductivity (C.E)	0.45 dS/m	Low salinity
Nitrate (NO3-N)	12.83 ppm	Low nitrogen
Phosphorus (P)	13.40 ppm	High phosphorus
Potassium (K)	182.68 ppm	High potassium
Organic Matter (MO)	2.13%	Moderate organic matter

**Table 3:** Durum wheat cultivars used in the experiment.

Cultivar	Year of Registration	Pedigree
Karim	1985	Bittern ‘S’ «JO’S’. AA”:S’//FG’S’»
Nassira	2003	INRA Selection on CIMMYT EII, 12 TA14/BD3//Isly # CF41530–1548
Faraj	2007	Hybrid Nassira, Qarmal, Lahn (ICARDA)
Luiza	2011	RASCON_39/TILO_1
Itri	2017	RISSA/GAN//POHO_1/3/PLATA_3//CREX/ALLA/x Karim

**Table 4:** Impact of nitrogen doses and seeding rates on wheat yield, biomass, and protein content across different treatment combinations.

Nitrogen Dose	Seed Rate	Mean Yield (kg/ha)	Standard Deviation	Mean Biomass (kg/ha)	Standard Deviation	Mean Protein %	Standard Deviation
N1	S1	2841 ^ab^	335	9800 ^a^	1651	16.91 ^ab^	2.55
	S2	2622 ^abc^	614	9017 ^ab^	1197	15.26 ^b^	2.27
	S3	2761 ^abc^	513	9367 ^ab^	1268	17.82 ^ab^	1.45
Mean		2741	487	9395	1372	16.66	2.09
N2	S1	2279 ^abc^	443	8825 ^ab^	1075	16.53 ^ab^	1.69
	S2	2978 ^a^	1035	9742 ^a^	2742	17.16 ^ab^	1.74
	S3	2008 ^c^	462	7508 ^b^	1122	17.79 ^ab^	0.91
Mean		2422	647	8692	1646	17.16	1.45
N3	S1	2349 ^abc^	453	7542 ^b^	1216	19.12 ^a^	3.24
	S2	2203 ^bc^	363	7450 ^b^	696	17.86 ^ab^	3.16
	S3	2470 ^abc^	190	8233 ^ab^	1027	17.76 ^ab^	1.47
Mean		2341	335	7742	980	18.25	2.62
Source		F-Value (Yield)	*p*-Value (Yield)	F-Value (Biomass)	*p*-Value (Biomass)	F-Value (Protein)	*p*-Value (Protein)
Nitrogen Dose		4.65	0.012 *	9.92	0.000 ***	4.11	0.02 *
Seed Rate		0.92	0.402 ns	0.62	0.539 ns	1.79	0.173 ns
Nitrogen × Seed Rate		4.38	0.003 **	3.53	0.01 *	1.88	0.122 ns

Notes: ANOVA: * = *p* ≤ 0.05, ** = *p* ≤ 0.01, *** = *p* ≤ 0.001, and ns = not significant. Means with identical letters are not significantly different at the 95% confidence interval (Tukey method).

**Table 5:** Effects of nitrogen doses and seeding rates on Normalized Difference Vegetation Index (NDVI) measurements at three key growth stages of wheat.

Nitrogen Dose	Seed Rate	NDVI 1 Mean	Standard Deviation	NDVI 2 Mean	Standard Deviation	NDVI 3 Mean	Standard Deviation
N1	S1	0.834 ^a^	0.031	0.818 ^ab^	0.045	0.813 ^a^	0.034
	S2	0.817 ^a^	0.038	0.807 ^ab^	0.044	0.81 ^a^	0.044
	S3	0.84 ^a^	0.028	0.857 ^a^	0.026	0.781 ^a^	0.074
Mean		0.830	0.032	0.827	0.038	0.801	0.051
N2	S1	0.815 ^a^	0.022	0.796 ^ab^	0.029	0.767 ^a^	0.06
	S2	0.818 ^a^	0.06	0.802 ^ab^	0.067	0.784 ^a^	0.059
	S3	0.805 ^ab^	0.028	0.793 ^b^	0.037	0.8 ^a^	0.027
Mean		0.813	0.037	0.797	0.044	0.784	0.049
N3	S1	0.757 ^b^	0.04	0.772 ^b^	0.021	0.761 ^a^	0.036
	S2	0.806 ^ab^	0.023	0.788 ^b^	0.036	0.772 ^a^	0.037
	S3	0.799 ^ab^	0.053	0.777 ^b^	0.066	0.779 ^a^	0.039
Mean		0.787	0.039	0.779	0.041	0.771	0.037
Source		F-Value (NDVI 1)	*p*-Value (NDVI 1)	F-Value (NDVI 2)	*p*-Value (NDVI 2)	F-Value (NDVI 3)	*p*-Value (NDVI 3)
Nitrogen Dose		9.64	0.000 ***	9.42	0.000 ***	3.09	0.051 ns
Seed Rate		1.03	0.361 ns	0.76	0.472 ns	0.24	0.785 ns
Nitrogen × Seed Rate		2.56	0.045 *	1.61	0.181 ns	1.3	0.278 ns

Notes: ANOVA: * = *p* ≤ 0.05, *** = *p* ≤ 0.001, and ns = not significant. Means with identical letters are not significantly different at the 95% confidence interval (Tukey method).

**Table 6:** Influence of nitrogen dose and seed rate on SPAD chlorophyll content at key developmental stages of wheat.

Nitrogen Dose	Seed Rate	SPAD 1 Mean	Standard Deviation	SPAD 2 Mean	Standard Deviation	SPAD 3 Mean	Standard Deviation
N1	S1	50.03 ^ab^	1.78	49.88 ^a^	2.31	45.98 ^ab^	4.76
	S2	51.97 ^ab^	2.25	50.62 ^a^	1.63	48.98 ^a^	4.92
	S3	52.47 ^a^	2.81	50.25 ^a^	2.5	46.23 ^ab^	6.57
Mean		51.49	2.28	50.25	2.15	47.06	5.42
N2	S1	49.73 ^ab^	2.93	50.69 ^a^	2.94	46.79 ^ab^	3.12
	S2	51.08 ^ab^	2.02	48.92 ^a^	2.42	42.11 ^b^	4.42
	S3	52.15 ^ab^	2.32	49.21 ^a^	2.09	47.75 ^ab^	5.19
Mean		50.99	2.42	49.61	2.48	45.55	4.24
N3	S1	50.9 ^ab^	2.12	50.79 ^a^	2.79	48.02 ^ab^	3.66
	S2	48.81 ^b^	3.04	49.05 ^a^	2.96	45.12 ^ab^	3.7
	S3	50.12 ^ab^	2.02	49.51 ^a^	3.08	41.7 ^b^	4.72
Mean		49.94	2.39	49.78	2.94	44.95	4.03
Source		F-Value (SPAD 1)	*p*-Value (SPAD 1)	F-Value (SPAD 2)	*p*-Value (SPAD 2)	F-Value (SPAD 3)	*p*-Value (SPAD 3)
Nitrogen Dose		3.22	0.045 *	0.5	0.608 ns	1.65	0.198 ns
Seed Rate		2.53	0.086 ns	1.14	0.324 ns	1.21	0.304 ns
Nitrogen × Seed Rate		2.4	0.056 ns	0.84	0.507 ns	4.43	0.003 **

Notes: ANOVA: * = *p* ≤ 0.05, ** = *p* ≤ 0.01 and ns = not significant. Means with identical letters are not significantly different at the 95% confidence interval (Tukey method).

**Table 7:** Effects of nitrogen dose and seed rate on canopy temperature dynamics during wheat growth stages.

Nitrogen Dose	Seed Rate	CT 1 Mean	Standard Deviation	CT 2 Mean	Standard Deviation	CT 3 Mean	Standard Deviation
N1	S1	20.95 ^a^	0.60	24.05 ^a^	1.08	23.58 ^a^	1.64
	S2	20.72 ^ab^	0.22	23.64 ^a^	0.84	23.63 ^a^	0.80
	S3	19.72 ^c^	0.85	22.92 ^a^	1.42	23.60 ^a^	1.01
Mean		20.46	0.56	23.53	1.11	23.60	1.15
N2	S1	20.26 ^abc^	0.61	23.16 ^a^	0.62	23.93 ^a^	0.85
	S2	20.00 ^bc^	0.45	23.61 ^a^	0.57	23.59 ^a^	1.05
	S3	20.48 ^abc^	0.55	23.49 ^a^	0.85	24.11 ^a^	0.93
Mean		20.24	0.54	23.42	0.68	23.88	0.94
N3	S1	20.24 ^abc^	0.42	23.07 ^a^	0.67	23.78 ^a^	0.89
	S2	20.03 ^bc^	0.38	23.04 ^a^	0.70	24.00 ^a^	0.81
	S3	20.54 ^ab^	0.62	23.06 ^a^	0.66	23.90 ^a^	0.87
Mean		20.27	0.47	23.05	0.67	23.89	0.86
Source		F-Value (CT 1)	*p*-Value (CT 1)	F-Value (CT 2)	*p*-Value (CT 2)	F-Value (CT 3)	*p*-Value (CT 3)
Nitrogen Dose		1.45	0.24 ns	2.54	0.085 ns	0.78	0.464 ns
Seed Rate		1.84	0.166 ns	1.02	0.365 ns	0.14	0.869 ns
Nitrogen × Seed Rate		8.26	0.000 ***	2.07	0.093 ns	0.33	0.856 ns

Notes: ANOVA: *** = *p* ≤ 0.001, and ns = not significant. Means with identical letters are not significantly different at the 95% confidence interval (Tukey method).

## Data Availability

The data presented in this study are available on request from the corresponding author. The data are not publicly available due to ongoing analyses and intended use in future publications.
